# Comorbidities and Monitoring in Patients with Chronic Hepatitis B on Nucleos(t)ide Analogues Attending a Tertiary Hospital in Malaysia, Southeast Asia: A Critical Perspective

**DOI:** 10.21315/mjms2024.31.4.12

**Published:** 2024-08-27

**Authors:** Kizito Eneye Bello, Itodo Janefrancis Kelechi, Zakari A. David, Adejo Patience Omebije, Rafidah Hanim Shueb, Nazri Mustaffa

**Affiliations:** 1 Department of Medical Microbiology and Parasitology, School of Medical Sciences, Universiti Sains Malaysia, Kelantan, Malaysia; 2 Kogi State University (Prince Abubakar Audu University) Anyigba, Kogi State, Nigeria; 3 Institute for Research in Molecular Medicine (INFORMM), Universiti Sains Malaysia, Kelantan, Malaysia; 4 Department of Medicine, School of Medical Sciences, Universiti Sains Malaysia, Kelantan, Malaysia; 5 Hospital Universiti Sains Malaysia, Kelantan, Malaysia

**Keywords:** chronic hepatitis B, comorbidities, nucleos(t)ide, treatment, monitoring, Malaysia

## Abstract

**Background:**

Chronic hepatitis B (CHB) is a significant global public health concern in Malaysia. It is a potentially life-threatening liver disease caused by the hepatitis B virus (HBV), which can lead to long-term complications such as cirrhosis, liver failure and hepatocellular carcinoma. In managing CHB, nucleos(t)ide analogues (NAs) have become the preferred treatment due to their ability to suppress viral replication and prevent disease progression. The question of liver-associated comorbidities related to patients with CHB on NAs remains unresolved in Malaysia despite the impending burden of CHB in the country. This study intends to address this and related aspects.

**Method:**

We assessed 136 CHB patients on NAs in one centre, the Hospital Universiti Sains Malaysia. Demographic and epidemiological data on the treatment, concomitant disease and monitoring strategies were collected and analysed.

**Result:**

Patients on NAs aged 50 years old–70 years old had the highest proportion of CHB (45.59%), with males representing 61.03% of that age group. There was a statistical significance in CHB acquisition and presence of comorbidities at *P >* 0.005. Our cohort displayed seven comorbidities (diabetes, obesity, rheumatoid diseases, renal impairment, spontaneous bacterial peritonitis, hypertension, non-hepatocellular malignancies and carcinoma); hypertension had the highest incidence (69.12%), while renal impairment had the lowest incidence (8.09%). Whole blood count, liver function and creatinine tests were the major monitoring tests used in over 90% of the cohort compared to viral load (6.1%).

**Conclusion:**

Diabetes, hypertension and obesity were independent risk factors for acquiring liver cirrhosis and hepatocellular carcinoma. Malaysian CHB patients treated with NAs have several comorbidities that could affect disease outcomes. Therefore, careful monitoring is required.

## Introduction

The hepatitis B virus (HBV) is a worldwide public health concern (1). Although the incidence and morbidity of chronic HBV infections have been dropping because of extensive vaccination programmes, the number of HBV cases is not significantly decreasing, due to population growth (2–4). Globally, and in Malaysia, chronic hepatitis B (CHB) is a serious public health issue (5). HBV is the source of this potentially fatal liver condition, which can result in long-term consequences such as cirrhosis, liver failure and hepatocellular cancer (6). Nucleos(t)ide analogues (NAs), which have the potential to inhibit viral replication and stop disease development, have emerged as the primary treatment for CHB (7). It is projected that, soon, a rise in morbidity and mortality will result from both the active chronic HBV progression stages and the ageing of CHB patients, regardless of whether they are HBeAg-positive or -negative (8). Creating NAs fundamental to treating this illness (9, 10).

Comorbid conditions are frequently present in CHB patients, complicating management and necessitating close observation. In patients with CHB, comorbidities such as diabetes mellitus, hypertension, dyslipidemia and renal impairment are rather common (11). The choice and efficacy of antiviral therapy can be affected by several comorbidities, which can also affect the general health of CHB patients (11). The successful lifelong suppression of HBV and its replications has been frequently demonstrated via NAs (12). The latter enhances the clinical outcomes and predisposes them to several comorbidities in CHB patients (12). Pegylated interferon alpha, an alternative treatment for CHB patients, has not yet shown a better tolerance and safety profile than NAs. They are the only available therapeutic option for several patient populations, including those with severe cirrhosis, immunosuppressive indications or extrahepatic symptoms (13).

Thus, NAs are used to treat the great majority of CHB patients. Several NAs, including telbivudine and lamivudine, show a low genomic barrier to HBV resistance, despite being licensed for use in CHB (14). Entecavir (ETV) and tenofovir derivatives are the only NAs indicated for CHB patients who have never previously taken NAs and have high NA resistance (15). Long-term management options include therapeutic treatment with ETV, tenofovir disoproxil fumarate (TDF). It has been repeatedly shown that NAs effectively limit HBV replication over the long term, improving all clinical outcomes for CHB patients (16). Interferon alpha, an alternate treatment for CHB patients, has not yet shown a higher level of tolerance and safety than NAs; it is also not cost-effective and has several side effects (17).

Additionally, it seldom results in HBV surface antigen (HBsAg) protein clearance or a ‘functional cure’, but they are the only treatment choice for several patient populations, especially those with severe cirrhosis, immunosuppressive conditions and other symptoms of CHB (18). Usually, therapy is given as a one off and necessitates careful long-term monitoring to prevent relapse. When treatment begins, other medical conditions may already be present in the elderly CHB patient group or may develop later. Because of this, those with current CHB and who take NAs are more likely to have concomitant disorders and require extra medication (19).

It is suggested that CHB patients receiving NAs undergo baseline and continuous on-therapy laboratory testing emphasising renal function. Based mainly on long-term ETV and TDF trials, it is widely thought that long-term NA treatment has little impact on bone mineral density and renal function. However, there have been reports of renal tubulopathy, reduced renal function and perhaps Fanconi syndrome among TDF users (20).

CHB patients are advised to switch from TDF to ETV or tenofovir alafenamide fumarate (TAF) and select those regimens over TDF if they already have renal or bone problems or are at a high risk of developing them. The only suggested treatment for people who have previously taken lamivudine is TAF (9, 17, 21 ).

TAF, on the other hand, has a safer profile than TDF in renal and bone laboratory markers, according to recent phase III trials (22). CHB patients are advised to switch from TDF to ETV or TAF and select those regimens over TDF if they already have renal or bone problems or are at a high risk of developing them. The only suggested treatment for people who have previously taken lamivudine is TAF (23).

The management of CHB and its associated comorbidities in tertiary hospitals, where complex cases are frequently referred, necessitates a multidisciplinary approach involving hepatologists, infectious disease experts, endocrinologists, cardiologists, nephrologists and other pertinent healthcare professionals (24, 25). Monitoring is crucial in handling patients with CHB on NAs. Regular monitoring enables healthcare professionals to evaluate treatment effectiveness, identify potential side effects, and modify therapy as necessary (26). The HBV viral load, renal function, glucose levels, lipid profile, blood pressure and liver function tests (such as alanine aminotransferase and bilirubin levels) are important factors that need to be tracked. Additionally, to evaluate liver fibrosis and check for hepatocellular carcinoma, imaging modalities like ultrasonography or fibroscans may be used (27).

It is critical to comprehend the frequency of comorbidities and the monitoring strategies and techniques in a tertiary hospital in Malaysia, a nation with a substantial burden of CHB (16, 28). With the aid of this information, healthcare professionals can create individualised plans for managing patients with CHB and comorbidities that will enhance patient outcomes and lessen the burden of illness. Considering the difficulties, we decided to conduct a study with the following goals to evaluate the monitoring schemes in a real-world setting at a gastroenterology clinic in a tertiary teaching hospital in Malaysia (Hospital Universiti Sains Malaysia) and examine the occurrence of other medical conditions aside from HBV and their relevance in HBV disease progression and outcomes.

This study aims to contribute to the development of evidence-based guidelines and recommendations for the management of CHB and comorbidities, ultimately improving the quality of care given to these patients. Hospital Universiti Sains Malaysia, a national referral and teaching hospital with a bed capacity of 500, is located at Kubang Kerian, in Kota Bharu District of Kelantan, Malaysia, as the Gastroenterology Clinic in the Department of Medical Microbiology and Parasitology, School of Medical Sciences, Universiti Sains Malaysia (29, 30). The hospital provides health care services to around 50,000 to 80,000 patients, including the student community, every year. This study assessed outpatients referred for laboratory examination who were 18 years old and older. The hospital lies at 6.099 N and 102.28 E, with a tropical climate (29).

## Method

### Patient Population

A retrospective study was conducted in Hospital Sains Malaysia, a public tertiary academic institute in Kelantan, Eastern Malaysia. We included 226 CHB patients who received NAs between June 2021 and July 2022 while visiting the gastroenterology clinics at Hospital Universiti Sains Malaysia. All patients met the requirements for NA therapy under the national criteria. All patients had persistent HBV infection for at least 6 months before starting NA medication. Exclusion criteria included the presence of concurrent hepatitis D, hepatitis C or HIV infections. All patients received the recommended dosage of each medication.

Relevant demographic, clinical and pharmacologic data from the patient records and information on co-occurring medical conditions were extracted. Age, gender, ethnicity, use of alcohol and tobacco, history of liver scarring, stiffness, cirrhosis at the beginning of NA therapy and HBV disease progression into hepatocellular carcinoma (HCC) during NA therapy, were some of the data recorded.

### Statistical Analysis

The statistical software programmes GraphPad and SPSS were used to carry out the analysis. The mean, standard deviation (SD) or median (range) values were used to express quantitative data. The chi-squared test was utilised for categorical data. It was decided that a probability value of 0.05 was statistically significant (31).

## Results

**Figure 1:**
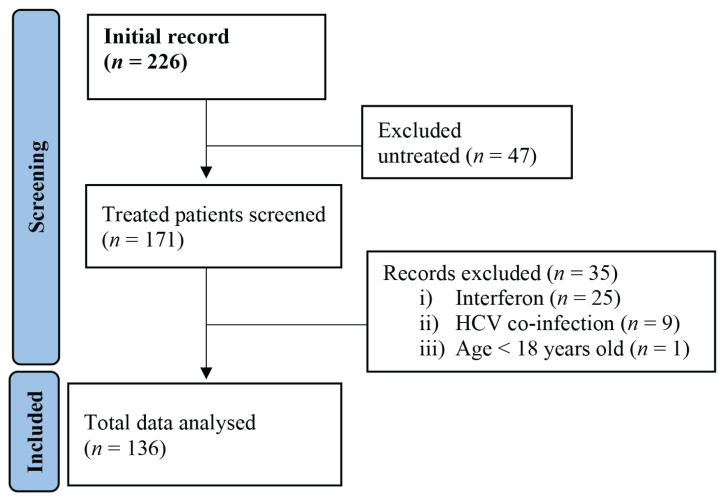
Summary of the inclusion process for patients with CHB and treated with NAs

From the hospital’s health records, we retrieved information on all 226 CHB virus patients covering the period of interest. After excluding naïve participants (untreated), 171 patients remained. We assessed medical records from these patients and excluded a further 35 patients (those on interferon treatment co-infected with hepatitis C virus and those under 18 years old of age), as shown in Figure 1. Medical records from the remaining 136 chronic patients were selected and analysed for this study.

The demographic characteristics of the enrolled participants are shown in Table 1. About 65% of the patients were older than 50 years old. The male-to-female ratio was approximately 2:1. Most recruited patients were Malay (*n* = 80, 58.82%). Alcohol consumption was insignificant to the acquisition of other comorbidities at *P* > 0.005. Approximately, 98% (134) of the examined participants did not use alcohol. The proportion without multiple sexual partners (69.85%) was twice that of those with multiple sexual partners (30.15%). There was a low proportion of current smokers compared to those with a history of smoking. Non-smokers (54.4%) were three times more numerous than current smokers (17.65%) and twice as numerous as patients with a history of smoking (27.94%). Smoking status was statistically significant with respect to the presence and acquisition of other comorbidities, as shown in Table 1. A higher proportion of the participants were on lamivudine (46.32%) compared to other NAs. The combination of tenofovir and ETV therapy had a low proportion (13.97%) compared to other nucleoside monotherapy. A high level of lamivudine resistance (31.62%) was present among the recruited CHB patients on NAs. The proportion of hepatocellular carcinoma (12.5%) among the participants was relatively significant, as liver cancer occurs in one of every seven candidates. There was a high proportion of candidates with liver cirrhosis (33.09%) compared to those with a history of organ transplant (1.47%) or surgery (8.82%), as shown in Table 1.

There were seven common comorbidities within our sample, as represented in Table 2. The most commonly encountered comorbidities in our study were hypertension (69.12%), diabetes mellitus (36.03%), obesity (13.24%), rheumatologic diseases (3.68%%), renal impairment (8.09%), non-hepatocellular carcinomas (16.91%) and arthritis (6.62%).

The proportion of participants with spontaneous bacterial peritonitis (SBP) was high (13.97%). Nine out of the 19 examined SBP patients were receiving ETV, while three were on tenofovir and seven were on lamivudine. Interestingly, six out of nine patients with arthritis were on lamivudine treatment and three were on tenofovir treatment. There was a correlation between the choice of treatment analogues and arthritis. Another interesting finding was the proportion of non-hepatocellular malignancies and carcinomas on lamivudine therapy. Sixteen out of 23 non-hepatocellular malignancies and carcinomas in our recruited cohort were on lamivudine therapy. The renal impairment was the lowest concurrent condition observed in our cohort (8.09%).

The European Association for the Study of Liver advises its members to schedule at least two medical appointments yearly (32). Table 3 shows the typical tests used to assess the effectiveness and safety of antiviral regimens, the percentage of patients in our cohort that have these tests and the frequency of the tests.

While liver function tests were performed in over 90% of our cohort, hepatitis B DNA concentration was found 67% of our recruited participants. Other standard laboratory tests include whole blood count, viral load, liver function tests, creatinine and liver biopsy. At least 43% of the patients recruited underwent these procedures twice a year (every 6 months). Liver biopsies and ultrasounds are performed between 8 and 20 times a year. Forty-three per cent of individuals receiving tenofovir had their whole blood count assessed. In instances with cirrhosis, the percentage of patients who received HCC surveillance, at least with ultrasonography, did not differ significantly (*P* > 0.005). Patients with cirrhosis and kidney impairment, respectively, had more frequently performed liver function tests (8.2 ± 2.4) and liver biopsies (6.9 ± 5.1). Patients with medium-high risk underwent cirrhosis surveillance more regularly than patients at low HCC risk (frequency: 6.1 ± 2.4 versus 7.8 ± 6.9 months, *P* < 0.005).

**Figure 2:**
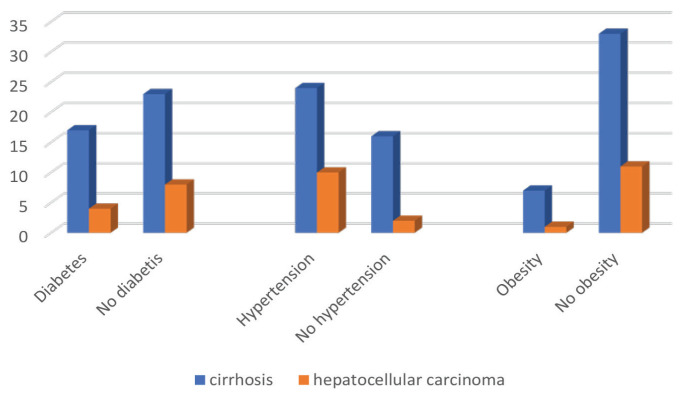
Number of treated CHBV patients with liver cirrhosis and hepatocellular carcinoma categorised by other comorbidities

There was a trend for cirrhosis to be more commonly present in the non-diabetic cohort than in the diabetic cohort. HCC was found to have developed more often in non-diabetic CHB patients than in those with diabetes mellitus (11% versus 3%, *P* = 0.022) (Figure 2). In addition, patients with HCC development were significantly more abundant in the hypertensive cohort than those without hypertension. The presence of obesity was not significant in the acquisition of cirrhosis and HCC within our cohort.

## Discussion

Since over 80% of CHB patients receiving NA treatment were close to or older than 50 years old, our study indicates that this population is ageing. They could therefore display a few non-hepatic illnesses co-infecting a patient. These concomitant medical conditions could significantly affect the development and natural course of liver disease and present major management concerns.

In our study, sex was a significant factor among CHB patients on NAs for the acquisition of other co-existing comorbidities. Our findings are consistent with those of a previous report (20). CHB patients seen and treated in Malaysia comprise four major ethnic groups (Malay, Chinese, Indian and others). The largest ethnic group is the Malay, who tended to be older in our sample; there are several theories for the high proportion of aged CHB patients, but it could be due to the higher population index of the Malay ethnic group in the Malaysian peninsula where the study was carried out, as Malay is the predominant ethnicity in Kelantan, where the tertiary teaching hospital is located (33–35).

A notable trend is that CHB in Malaysia is disappearing in the younger population. Introducing a preventive vaccine has reduced the associated morbidity of CHB infection in Malaysia among young people (less than 40 years old), after the introduction of a nationwide vaccination programme in 1989 (36). While HBV incidence is decreasing in Malaysia, the morbidity of CHB will not disappear soon. It is anticipated that there will be a growing number of CHB patients receiving NAs. Interestingly, there is a smaller but growing subgroup in our study that includes mostly younger CHB patients who were not vaccinated or acquired ‘vaccine escape’ variants of HBV. These findings are consistent with other reports (16, 18, 27, 37–41).

Alcohol consumption was not statistically significant to the acquisition of comorbidities in our study, while the cultural and religious background of the major ethnic group contributed significantly to the low incidence of alcoholic intake in our study, as Islam forbids alcohol consumption. The findings of our study are consistent with other reports that cultural norms and moral values influence the morbidity of disease (38–41). Apart from alcohol intake, men are allowed to have multiple wives within the confines of Islamic marital rights. Hence, the reason for the high incidence of multiple sexual partners is worth mentioning, in that the societal norms forbid a woman from having multiple sexual partners within the study area, which is one of the Islamic sultanates within the Malaysian peninsula. Our findings are consistent with other reports (41, 42).

The proportion of non-smokers was higher than those with a smoking history and current smokers. Though statistically significant, the reason for the high variation between non-smokers and current smokers is unclear. Smoking has been implicated as an influencing risk factor in the acquisition of HBV and other diseases (43, 44).

Due to the significant prevalence of lamivudine-resistant variants of HBV, ETV and Tenofovir regimens were predominantly utilised as NAs in Malaysia throughout the past 10 years (45). There appears to be a sizable subgroup of CHB cases that should be treated with tenofovir, given that almost half of our patients had experience of lamivudine and a quarter had proven lamivudine resistance (45). However, the existence of comorbidities may give rise to legitimate concerns about using tenofovir (46, 47).

Hepatocellular carcinoma had a low incidence (12.5%) among our cohorts compared to cirrhosis (33.09%). The probable reason for our cohort’s high cirrhosis incidence among HCCs is unclear, but can likely be attributed to the lamivudine experience of the cirrhosis and HCC cohort, as over 60% of HCC and over 75% of cirrhosis patients had experience with the lamivudine regimen. The findings of our study are consistent with other reports (14). Cirrhosis and HCC were statistically significant (*P* < 0.005) regarding the acquisition of other comorbidities. Cirrhosis and HCC alter the natural course of CHB, thereby compromising the cellular immunity of CHB patients. The latter influences the occurrence of opportunistic infections. The findings of our study are also consistent with the reports of other researchers (48–51).

Organ transplant and history of surgery were not significant contributors to comorbidities. Hypertension and diabetes had the highest estimate among other comorbidities associated with CHB patients on the NAs regimen. Most indigenous Malaysian cuisines are predominantly sugary, as in the Kelantan sultanate, which had a significant estimate of diabetes in Malaysia (52, 53). The high toll of hypertension could be associated with several factors; other comorbidities, other medical complications, feeding habits, and increased consumption of table salt have been established as factors predisposing patients towards the acquisition of hypertension. Local cuisine in Malaysia is prepared with a proportional addition of sugar and salt to add taste to the meal (54). Our findings are consistent with the report of Remali et al. (52) and Chow et al. (55) who reported a high prevalence of diabetes and hypertension in the Malaysian population, respectively. The presence of arthritis and rheumatoid disease is indicative that the disease spectra of the CHB are gradually transforming into a more novel clinical presentation. The occurrence of arthritis and rheumatoid disease can be associated with immunolocalising changes that were triggered with the initiation of the nucleos(t)ide regimens. This finding is consistent with those of previous reports (56–58).

The presence of SBP in our study triggers some concerns, as SBP in viral cirrhosis contributes to developing other liver diseases such as hepatocellular carcinoma and decompensated cirrhosis. The findings of our report are consistent with our previous report (6), where we reported the prevalence of SBP in hepatitis viral cirrhosis in Asia. Our study reported the presence of non-hepatocellular carcinomas and malignancies. Despite the low incidence of these carcinomas and malignancies, it indicates recurrent hepatitis B reactivation among CHB. The latter could be due to the highly resistant progeny of HBV replication (59). Obesity has been confirmed by previous reports to be an independent risk factor for several diseases. The presence of obesity among our study cohort is suggestive of multiple potential comorbidities within the cohort (60).

The monitoring strategies reported in our study were consistent with national guidelines. The only variation was in the frequencies of some of the tests. Viral load tests were performed less frequently than the universal standards, probably due to the high cost of DNA quantification tests for HBV and the lack of government subsidies for viral load testing in Malaysian public hospitals. Whole blood count and liver function tests are routine tests for monitoring CHB progression (61). A liver biopsy was performed to monitor the disease progression into liver cirrhosis (62). In our study, the correlation between obesity, diabetes and hypertension and the emergence of cirrhosis and hepatocellular carcinoma had the most significant impact on the outcomes of liver disease caused by the presence of comorbidities.

It has been hypothesised that diabetes and obesity have particularly harmful effects on the liver, working in concert with the underlying chronic HBV infection to cause HCC. Diabetes has not been identified as an independent risk factor for HCC in prior research (63). The development of liver cirrhosis, hepatocellular carcinoma and hypertension were related. Recent studies (64) have also highlighted this link, underlining the importance of CHB patients with diabetes maintaining a high level of alertness to spot any early indications of the development of cirrhosis and HCC.

## Conclusion

In conclusion, NAs are Malaysia’s only long-term maintenance medication for the treatment of CHB patients. Such patients need careful management because many are more than 50 years old and there are frequent associations with several comorbidities. These comorbid conditions may affect how the disease develops over its normal course. Consequently, careful management of any existing comorbidities and diligent monitoring over an extended period are required.

## Figures and Tables

**Table 1:** Demographic and clinical characteristics of CHB patients on NAs

Characteristics	No. examined	Proportion (%)	Pearson’s chi-squared test	*P*-value
Age (years old)			2.993	0.038
18–30	7	5.15		
31–50	36	26.47		
51–70	62	45.59		
> 70	31	22.80		
Gender			2.728	0.043
Male	83	61.03		
Female	54	39.71		
Ethnicity			2.532	
Malay	80	58.82		
Chinese	46	33.82		
Indian	8	5.88		
Other	0	0.00		
Alcoholic consumption			2.105	0.057
Yes	2	1.47		
No	134	98.53		
Multiple sexual partners			2.056	0.078
Yes	41	30.15		
No	95	69.85		
Smoking			1.098	0.021
Never	74	54.41		
Former	38	27.94		
Current	24	17.65		
Antiviral treatment Lamivudine			2.65	0.077
Yes	63	46.32		
No	73	53.68		
Tenofovir			2.11	0.05
Yes	39	28.68		
No	97	71.32		
Entecavir			2.109	0.058
Yes	29	21.32		
No	107	78.68		
Lamivudine resistance			2.11	0.05
Yes	43	31.62		
No	93	68.38		
Hepatocellular carcinoma			2.73	0.047
Yes	17	12.5		
No	119	87.5		
Liver cirrhosis			2.46	0.045
Yes	45	33.09		
No	91	66.91		
Major surgery (organ transplant)			2.33	0.158
Yes	2	1.47		
No	134	98.53		
Other minor surgery			2.27	0.082
Yes	12	8.82		
No	124	91.18		

**Table 2:** Common comorbidities in CHB patients with NAs

Comorbidity	Number examined	Proportion (%)
Diabetes	49	36.03
Obesity	18	13.24
Rheumatoid disease	12	8.82
Renal impairment	11	8.09
Non-hepatocellular malignancies and carcinomas	23	16.91
Spontaneous bacterial peritonitis	19	13.97
Hypertension	94	69.12

**Table 3:** Tests used for monitoring disease progression and treatment effectiveness of CHBV on NAs

Tests	Proportion of patients (%)	Frequency of test + standard deviation	Frequency range	*P*-value	Confidence interval
Whole blood count	92	7.2 ± 2.4	3–18	0.173	2.1–23.5
Viral load	67	6.1 ± 3.2	2–10	0.083	1.2–13.8
Liver function test	99	10.3 ± 5.8	2–24	0.059	1.1–10.4
Creatinine	94	6.8 ± 2.6	1–15	0.085	1.2–13.7
Liver biopsy/ultrasound	43	15.9 ± 6.9	4–15	0.092	1.9–21.9
